# Supplementary material for: Hematological and Biochemical changes in Schistosoma mansoni infected patients at Haik Primary Hospital, North-East Ethiopia: A comparative cross-sectional study
Source: PLoS Negl Trop Dis. 2022 Aug 30;16(8):e0010728. doi: 10.1371/journal.pntd.0010728 (PMC9467371; doi:10.1371/journal.pntd.0010728)
Supplement: S1 Questionnaire — (DOCX) [file pntd.0010728.s001.docx]

**Questionnaire for assessing the Hematological and Biochemical changes in *Schistosoma mansoni* infected patients at Haik Primary Hospital, North-East Ethiopia: A comparative cross-sectional study**

1. **Schistosoma mansoni infected adult patients**
2. **Socio-demographic variables**

| **S.NO_** | **Question to be asked** | **Possible answers** |
| --- | --- | --- |
|  | ID | ___________________ |
|  | Age | ___________________ |
|  | Sex | 1. Male B. female |
|  | Marital status | 1. Single B. Married C. Divorced   D. Widowed |
|  | Residence | 1. Urban B. Rural |
|  | Educational status | 1. Illiterate B. primary C. Secondary   D. College and above |
|  | Occupation | 1. Farmer B. House wife C. Gov.t employee D. Private |

1. **Hematological parameters and Biochemical parameters**

| **S/number** | **Parameters** | **Result** |
| --- | --- | --- |
|  | **ID___________________ Age___________ Sex___________** | |
| 1 | WBC (10^3^µL) |  |
| 2 | Neutrophile (10^3^µL) |  |
| 3 | Lymphocyte (10^3^µL) |  |
| 4 | Monocyte (10^3^µL) |  |
| 5 | Eosinophile (10^3^µL) |  |
| 6 | Basophil(10^3^µL) |  |
| 7 | RBC (10^6^µL) |  |
| 8 | Hgb (g/dL) |  |
| 9 | HCT (%) |  |
| 10 | MCV (fL) |  |
| 11 | MCH (pg) |  |
| 12 | MCHC (g/dL) |  |
| 13 | RDW (fL) |  |
| 14 | PT (10^3^µL) |  |
| 15 | MPV (fL) |  |
| 16 | PDW (fL) |  |
| 17 | ESR (mm) |  |
| 18 | ALT (IU/L) |  |
| 19 | AST (IU/L) |  |
| 20 | TP (g/dL) |  |
| 21 | ALB (g/dL) |  |
| 22 | TBIL (mg/dL) |  |
| 23 | DBIL (mg/dL) |  |
| 24 | ALP(IU/L) |  |
| 25 | TG (mg/dL) |  |
| 26 | TC (mg/dL) |  |

**Questionnaire for assessing the Hematological and Biochemical changes in *Schistosoma mansoni* infected patients at Haik Primary Hospital, North-East Ethiopia: A comparative cross-sectional study**

1. **Apparently healthy controls**
2. **Socio-demographic variables**

| **S.NO_** | **Question to be asked** | **Possible answers** |
| --- | --- | --- |
|  | ID | ___________________ |
|  | Age | ___________________ |
|  | Sex | 1. Male B. female |
|  | Marital status | 1. Single B. Married C. Divorced   D. Widowed |
|  | Residence | 1. Urban B. Rural |
|  | Educational status | 1. Illiterate B. primary C. Secondary   D. College and above |
|  | Occupation | 1. Farmer B. House wife C. Gov.t employee D. Private |

1. **Hematological parameters and Biochemical parameters**

| **S/number** | **Parameters** | **Result** |
| --- | --- | --- |
|  | **ID___________________ Age___________ Sex___________** | |
| 1 | WBC (10^3^µL) |  |
| 2 | Neutrophile (10^3^µL) |  |
| 3 | Lymphocyte (10^3^µL) |  |
| 4 | Monocyte (10^3^µL) |  |
| 5 | Eosinophile (10^3^µL) |  |
| 6 | Basophil(10^3^µL) |  |
| 7 | RBC (10^6^µL) |  |
| 8 | Hgb (g/dL) |  |
| 9 | HCT (%) |  |
| 10 | MCV (fL) |  |
| 11 | MCH (pg) |  |
| 12 | MCHC (g/dL) |  |
| 13 | RDW (fL) |  |
| 14 | PT (10^3^µL) |  |
| 15 | MPV (fL) |  |
| 16 | PDW (fL) |  |
| 17 | ESR (mm) |  |
| 18 | ALT (IU/L) |  |
| 19 | AST (IU/L) |  |
| 20 | TP (g/dL) |  |
| 21 | ALB (g/dL) |  |
| 22 | TBIL (mg/dL) |  |
| 23 | DBIL (mg/dL) |  |
| 24 | ALP(IU/L) |  |
| 25 | TG (mg/dL) |  |
| 26 | TC (mg/dL) |  |
