# Supplementary material for: Hematological and Biochemical changes in Schistosoma mansoni infected patients at Haik Primary Hospital, North-East Ethiopia: A comparative cross-sectional study
Source: PLoS Negl Trop Dis. 2022 Aug 30;16(8):e0010728. doi: 10.1371/journal.pntd.0010728 (PMC9467371; doi:10.1371/journal.pntd.0010728)
Supplement: Attachment — Submitted filename: PLOS NTD-2022.pdf [file pntd.0010728.s002.pdf]

Hematological and Biochemical changes in Schistosoma mansoni infected patients at Haik Primary Hospital, North-East Ethiopia: A comparative cross-sectional study

GENERAL CONSIDERATIONS AND SUGGESTIONS

Methodology

It is necessary to clarify the origin of the control group because it is a little strange to have a control group chosen within a hospital. The criterion used to determine the participants in this group has been based only on clinical information as mentioned in the item Socio-demographic characteristics and clinical data? Stool tests has been done? If yes, which method has been used?

The authors only mention (in the introduction) that schistosomiasis mansoni is endemic in Haik town, not revealing this endemicity index, an important factor in the analysis and determination of the groups.

A more detailed description of the area from which these patients originated was lacking. It is important to know if the region has *S. haematobium,* for example, and if this was also used as an exclusion criterion.

In the Item **"Stool sample collection and processing",** the last period about blood collection must be removed and added in the following item – **"Blood sample collection processing"**

Patients with splenomegaly were excluded and those with hepatomegaly, too? If these conditions are present in patients with severe forms of schistosomiasis, wouldn't it be interesting to keep them in the study group? An association could be made between the presence of these clinical forms and the hematological and biochemical indices analyzed.

Results.

The results are clear and well-presented between tables and graphs, I suggest, however, that you add the standard deviation in the graphs.

Discussion

The discussion in relation to hematological and biochemical indices is well founded, however I missed the approach on clinical and socio-demographic data.
There was a lack of an approach to public health using indicators from the region and also, the limitations found.
