# Supplementary material for: Hematological and Biochemical changes in Schistosoma mansoni infected patients at Haik Primary Hospital, North-East Ethiopia: A comparative cross-sectional study
Source: PLoS Negl Trop Dis. 2022 Aug 30;16(8):e0010728. doi: 10.1371/journal.pntd.0010728 (PMC9467371; doi:10.1371/journal.pntd.0010728)
Supplement: Attachment — Submitted filename: Point by point response to the editors and reviewers.docx [file pntd.0010728.s003.docx]

**Point by point response to the editors and reviewers**

We would like to pass our sincerely gratitude to the editor and the reviewers for reviewing our manuscript. We have revised the manuscript extensively in the light of the reviewers’ constructive suggestions. A point-by-point response is given below. We have also provided the clean version of the manuscript. All additional information added in the manuscript are marked by track changes. We hope that our revised manuscript will be suitable for publication in PLOS NTD.

**Reviewer #1**

“Systematic random sampling technique was employed to recruit the study participants at Haik Primary Hospital based on the inclusion criteria.” - how was the systematic random sampling conducted? Please elaborate.

**Response:** While reviewing the hospital registration book, on average two *S. mansoni* infected adult per-day are expected to be reported in the Hospital. Within 90 days of data collection period a total of 180 cases are expected to be reported. Then K value was calculated as 180/90=2. Finally, with lottery method sampling was begin in the second S. mansoni infected patient and the sampling process continued by systematically recruiting every other patient.

 “According to the rule of thumb recommended by van Voorhis and Morgan, 30 participants per group are required to detect real differences which could lead to about 80% power [21].” - We cannot rely on “rule of thumb”. Please describe appropriate sampling procedure. If random sampling from an adequate sample size was not done, then this should be described in limitations.

This would not also be probability sampling

**Response:** We use the rule of thumb as an initiation point to calculate the sample size. Even though their recommendation can give a power of 80%, we do not use the sample size they recommend. We increased it by 3 folds and we hope this is sufficient sample size the achieve our objectives. We applied systematic random sampling technique as explained in the previous response which is a one of the probability sampling techniques.

**Methods/data collection**

Both direct fecal smear and KK were used, but only KK was only used in quantification of ova. KK has higher sensitivity than DFS so please explain why KK was not used in determining if a sample is positive.

**Response:** thank you for your comment. Sorry we missed to included it in the document, but the fecal smear and KK were used for determining if a sample is positive or not. Now we have made the necessary corrections.

“A blood sample was collected from each study participant and healthy controls following standard operating procedures (SOPs) by trained laboratory technologists” – What are these SOPs? Please describe or at least put a reference or supplementary information

**Response:** We provide a reference.

3. Pictures of the processes are recommended

**Response:** thank you for your comment. Unfortunately, we did not have a captured picture of the process.

4. Patient-level (anonymised) were not provided

**Response:** All information pertaining to patients’ biodata that could be potentially used to identify the patient such as the patient names, were converted in to codes. Access to the original patient data was allowed only to the principal investigator and to the hospitals’ medical laboratory technologists.

5. Please include the tools (e.g., questionnaires) used.

**Response:** Now the questionnaire is included in the supplementary file.

**Ethical considerations**

Please provide documentation of ethics clearance - include clearance number and attach clearance

**Response:** We have provided the necessary information.

**Financial disclosure**

The study involved fieldwork and specimen processing, so it is strange that the authors declared not receiving funding. Please elaborate how the project expenses (whether in cash or in kind) were funded.

**Response:** Although there are no specified funds to the research, the reagents, equipment and some data collection related expenses were covered by Wollo University and we acknowledged them. Moreover, the authors of this manuscript are medical laboratory professionals with different specialties and carried out all the laboratory investigation will full of commitment and motivation.

Please describe how the data sets meet the requirements/assumptions for Kruskal-Wallis

**Response:** The variables are continuous variables. The distributions are not normally distributed as checked by Kolmogorov-Smirnov and Shapiro Wilk normality tests and the test should be non-parametric. It is four group (patient with light intensity, moderate intensity, heavy intensity of infection and healthy controls). If the number of groups to be compared are three and more, the Kruskal-Wallis H test is used to compare the variable.

**Please include CIs in all estimates.**

**Response:** thank you for your feedback. We have included the CI to the estimates.

The implications of the study are not clear. Please elaborate what will happen if we now know that schistosomiasis may result to biochemical changes. Please justify how we the conclusions have global relevance given the limited sample size.

**Response:** Knowing that schistosomiasis result in alteration of hematological and biochemical parameters will help as to get useful information about the different hematological and biochemical abnormalities in addition to the disease schistosomiasis itself. This study provides valuable information to clinician, academicians and other stakeholder who work on improving public health on how to design, adjust and implement effective treatment program to minimize schistosomiasis associated morbidity and mortality and maximize treatment impact. The finding of our study is an alert that treating schistosomiasis patients with praziquantel alone cannot improve the health of the patients, instead we have to consider and address the underlying diseases and provide the appropriate treatment. All these will lead to improved health status of schistosomiasis patients. And accelerate their recovery to healthy condition. The information will also alert the national and global health care providers to notice the impact of schistosomiasis and improve strategies to combat the disease.

Include site map

**Response:** We have included the site map.

Use indentation

**Response:** We act accordingly.

Please use line numbers

**Response:** Line number included.

Technical writing

Please elaborate what “Institution-based comparative cross-sectional study” means. Not a term I often encounter.

**Response:** Institution-based mean the study is conducted in health facilities/institution/ and it is not community based/survey. While comparative crossectional study is a a form of cross-sectional study where data on the two groups (in our case S. mansoni infected patients and healthy control) are collected at a point on time.

“Regular chat chewer” - What is chat? Please describe.

**Response:** Great apologize to type error committed. It is to mean ‘Khat’. Khat is a stimulant plant very common in East Africa and Yemen, which speeds up the messages going between the brain and the body.

Please improve English writing and proofread

**Response:** we have critically revised the whole document and act accordingly. We go thoroughly to entire document and amend the necessary spelling, grammar and vocabulary corrections.

“S. mansoni infected adults (≥18 years old) attending Haik Primary Hospital” - people do not attend hospitals

**Response:** Thank you for comment. We have replaced the word ‘attend’ by ‘visiting’.

It’s median, not “media”

**Response:** Sorry it is type error. We corrected it as Median.

“Three species of the genus Schistosoma (Schistosoma mansoni, Schistosoma hematobium, and Schistosoma japonicum) are responsible for the majority of Schistosomiasis in the World [2].” – I am not sure if there other genus which causes schistosomiasis?

**Responses:** There is no other genus which cause schistosomiasis. We have mentioned the genus Schistosma to distinguish it from other genera of class trematodes that cause diseases other than Schistosoma. This term is the most frequently used expressions for infectious diseases. In additions, indicating this as genus will help readers to avoid confusion especially to those who are not familiar with the parasites.

**Reviewer #2:**

The study objectives are well articulated and the study design is appropriate. The study population and the control groups are matching. The sample size is sufficient to draw conclusion and appropriate statistical method is used. Ethical considerations are clearly followed.

**Response:** Thank you for your feedback.

The presented result matches the analysis plan.  and completely presented.  comments for consideration are indicated in the last section.

**Response:** Thank you so much we have considered your valuable comments.

Conclusion and recommendation support the data presented. Authors had discussed the data is helpful and showed public health relevance.

**Response:** Thank you for your valuable feedback.

**Abstract**
Conclusion section “Therefore, screening of S. mansoni infected patients for various hematological and biochemical parameters and providing treatment to the underlying abnormalities is very crucial to avoid schistosomiasis associated morbidity and mortality of S. mansoni infected patients.” Should read “Therefore, screening of S. mansoni infected patients for various hematological and biochemical parameters and providing treatment to the underlying abnormalities is very crucial to avoid schistosomiasis associated morbidity and mortality.”

**Response:** Thank you for your valuable information. It is corrected as “Therefore, screening of S. mansoni infected patients for various hematological and biochemical parameters and providing treatment to the underlying abnormalities is very crucial to avoid schistosomiasis associated morbidity and mortality.”

**Introduction:**

Paragraph 3- remove “appendix”

**Response:** Removed.

**Method section:**

Since the sociodemographic characters collected are few, delete “Structured” in the questionnaire.
Statistical analysis: “Variables were expressed as median and interquartile range is” repeated

General: all scientific names should be italicized

**Response:** thank you. We made all the necessary corrections. The duplicates were removed and all scientific names were italicized.

**Result:**

Table 2- show the value for RBC

**Response:** Sorry for the error. We have included the value for RBC.

Under the “Prevalence and severity of anemia among S. mansoni infected patients” section correct “Moreover, 21.4% and 7.14% of females had mild and moderate anemic (Figure 1)” to “…….of females had mild and moderate anemia”

**Response:** Thank you for your comment. We have corrected it as per your suggestion.

Figure 1 needs revision to show the value for female’s study subjects and correction of “Non-amenic” to non-anemic

**Response:** Sorry again for the type error. It is corrected to non-anemic.

**Discussion:**

ESR section instead of “blood loose” it should read “blood loss”

**Response:** Thank you for the correction. We accepted you comment and act accordingly.

Confidence interval for intensity of infection should not be repeated in the discussion section.

**Response:** We act based on your comment.

The work is important to show healthcare points to be considered in schistosomiasis patients. The manuscript needs minor language editorial.

**Response:** Thank you for your valuable and constructive comments.

**Reviewer #3:**

Yes, the proposed objectives are in accordance with the suggested hypothesis;

Results are well presented, tables and graphs are clear, and statistical tests are well used.

**Response:** Thank you so much for you feedback.

I suggest adding the standard deviation to the graphs.

**Response:** Thank you for your suggestion. The graph is to indicate the prevalence of anemia and its severity. So, this is a categorical variable and we can’t add the standard deviation to the graph.

The results support the conclusions; however, limitations of the study were not described. Although the data are useful for a better understanding of the subject, there was no approach of this importance in relation to public health.

**Response:** Limitations are now added in the document.

No editorial suggestions

**Response:** Thank you for your positive comments.

**Reviewer #4:**

**Study Design and Methods:**

Study design or procedure: The description of the stages of the study was not shown. For instance, biological samples (stool and blood) were collected. Did the individuals undergo sample collection at any time during patient assistance at the primary hospital or only at the admission of the study? No details were informed.

**Response:** Thank you. We tried to describe the stage of the study in the method section. With regard to sample collection, we took the sample the study participants when they come to the hospital for seeking service. Both stool and blood samples were collected from the eligible participants following systematic random sampling technique. While samples for control group were collected in Dessie blood bank.

Ethical statement was not included in” Methods”.

**Response:** Ethical statement was included in the methods.

“Thus, a total of 180 (90 S. mansoni infected adults and 90 apparently healthy controls) were enrolled in the study”. There was no definition of “apparently healthy controls” described at “Eligibility criteria”.  Did any of the controls have a previous history of treated schistosomiasis? If yes, how long ago did they get the medication and proved treated before they were enrolled in the study?

**Response:** Apparently healthy controls (participants negative for *S. mansoni*, other parasitic infections and diseases that are reported to alter the hematological and biochemical parameters in individuals. None of the controls and cases have a history of previous schistosomiasis treatment.

Since other parasitic infections associated with anaemia may be a confounder, did the author excluded them before enrolling in the study? Is malaria an important issue in the study area? If yes, were both S. mansoni infected and controls investigated for malaria?

**Response:** Both *S. mansoni* infected patients and healthy controls were screened for malaria, Hookworm infection, and tirichuriasis and those infected with these parasites were excluded. Although malaria is not such an important issue in the study area, the study participants (both cases and controls) were screened for malaria. This is because malaria is issue in neighboring districts and participants might have travel history.

The data were presented mostly in tables that should be reviewed.

**Response:** We reviewed the data and made the necessary amendments.

**Tables and Figures:**

No footnotes were added to the respective Tables. Abbreviations were available only at the end of the text. It should be included in the footnotes under each table.

**Response:** Thank you. Footnotes are included in the revised document.

In Table 2 and 3, "normal", "Low" and "High" were not defined (laboratory reference values should be displayed in the footnote).

**Response:** Laboratory reference values are added.

Figure 1 showed a title without legend. Also, there is no identification of the ordinate (does it represent percentage?).

It is advisable to properly review it.

**Response:** Thank you for your comment we have worked on it as per your comment. Figure legends are available now. The coordinates are identified now the blue indicate the frequency and the blue chart indicate the percentage.

**Results:**
In "Intensity of infection, biochemical and hematological values": The WBC and RBC count was significantly lower in patients with moderate and heavy intensity of infection compared to patients with light intensity of infection and apparently healthy controls". The results described were not shown. (Table? statistical analysis?)

**Response:** Thank you for your comment. We assumed that it was sufficient to put the description only. Now we had provided the values in table form as per your suggestion.

**Discussion:**
An extensive literature review could improve the discussion of some issues. Example:
"Erythrocytic sedimentation rate (ESR) was significantly higher in S. mansoni infected patients than healthy controls. This finding was agreed with reports from Nigeria [23]. The elevation of ESR in S. mansoni patients might be due to blood loose as a result of bleeding during migration of worms in the intestine and consumption of blood by the worm". Should ESR be used to diagnosis and monitor inflammatory/fibrotic response? Was ESR elevated equally in men and women? May other comorbidities be confounders?

**Response:** Yes, ESR was elevated in S. mansoni infected patients and this test can be used as an indicative of S. mansoni infection. However, we can not say ESR is an accurate marker of the infection. It must be used along with other hematological parameters as well as other confounding situations must be considered and ruled out. The ESR was not elevated equally in male and female, here we put the sum of both male and female with elevated ESR. We also use different reference rage.

The use of hematological and biochemical markers in schistosomiasis management should be done in parallel with other tests (Image). For instance, intensity and extension of fibrotic liver response correlates with progression to chronic and advanced schistosomiasis. Although, authors propose the use of hematological and biochemical markers for screening the individuals infected with Schistosoma, the limitations of this strategy were not fully discussed.  The conclusions are weakly supported by the results. Also, added value of the study and implications of the results in the field were described.

**Response:** Thank you for your valuable comments. You are correct in addition to the hematological and biochemical parameters, imaging techniques especially in chronic infection is very important for monitoring and management of the disease. In our study we do not use the imaging techniques and we have put this as a limitation. Moreover, considering hematological and biochemical abnormalities in schistosomiasis patients, and screening of patients with hematological and/or biochemical abnormalities for S. mansoni infection in endemic areas is an important measure to reduce schistosomiasis associated morbidity and mortality as well as to improve the health of the society.

The data presentation should be completely reviewed (Tables and Figure).

**Response:** We have reviewed the document as per your comments.

This is a cross-sectional study that addresses both hematological and biochemical Schistosoma mansoni-induced alterations in infected individuals living in endemic área and attending medical assistance at a primary hospital in Haik town ( North-East Ethiopia). The study population includes adult individuals with schistosomiasis and a group of “apparently” healthy individuals as controls. The findings showed anemia and thrombocytopenia as the major laboratory-based disturbances in addition to abnormal biochemical results in Schistosoma infected individuals. Despite the lack of information about this particular geographical area, the results are not exactly new. Hematological and biochemical profiles of Schistosoma infected individuals were recently described in Amhara region in Ethiopia [Dessie et al, 2020]. Dessie et col. describe the hematological and biochemical profile of Schistosoma mansoni – infected individuals living in a study area with similar
altitude, population density and schistosomiasis prevalence as the present study [ Dessie N et al. J Trop Med.2020: 4083252. doi: 10.1155/2020/4083252; Bisetegn H et al. Trop Dis Travel Med Vaccines. 2021 ;7(1):30. doi: 10.1186/s40794-021-00156-0]. The importance of the studies on the subject is undeniable. To stablish the profile of Schistosoma-induced disease by using less expensive laboratory markers may help at least in two ways: to clarify aspects of Schistosoma infection pathological responses and improve disease management.  The last one is an important achievement in high-moderate endemic areas in low-income countries. However, the present manuscript brings a mild discussion on the main subject of the study which should be greatly improved.

**Response:** Thank you again for your comments. We have critically reviewed the manuscript; we go through extensive literature search and the implication of the study are now improved well. As you mentioned our main rationale for this study was lack of information in our geographical area. We also wanted to draw the attention of healthcare workers and other stakeholders towards *S. mansoni* infection and its association with the hematological and biochemical changes. We believe this would help health care workers to manage *S. mansoni* infected individuals appropriately and efficiently.
