# Supplementary material for: Hematological and Biochemical changes in Schistosoma mansoni infected patients at Haik Primary Hospital, North-East Ethiopia: A comparative cross-sectional study
Source: PLoS Negl Trop Dis. 2022 Aug 30;16(8):e0010728. doi: 10.1371/journal.pntd.0010728 (PMC9467371; doi:10.1371/journal.pntd.0010728)
Supplement: Attachment — Submitted filename: point by point response PLOS NTD.docx [file pntd.0010728.s004.docx]

**Point by point response to the editors and reviewers**

We would like to pass our sincerely gratitude to the editor and the reviewers for reviewing our manuscript. We have revised the manuscript extensively in the light of the reviewers’ constructive suggestions. A point-by-point response is given below. We have also provided the clean version of the manuscript. All additional information added in the manuscript are marked by track changes. We hope that our revised manuscript will be suitable for publication in PLOS NTD.

**Responses**
**Reviewer #1:** Thank you so much for all your valuable and constructive comment.

Can we properly calculate the sample size based on your research question and study design? I am not sure the article in Tutorials in Quantitative Methods for Psychology is an authoritative reference for sample size estimation.

**Response:** We use the rule of thumb as an initiation point to calculate the sample size. Even though their recommendation can give a power of 80%, we do not use the sample size they recommend. We increased it by 3 folds and we hoped this is more sufficient sample size to answer our research questions.

"The quality of the blood sample was maintained by collecting and processing it according to the229 standard operating procedures (SOPs) - the authors said that they had already provided a reference, but there is still none.

**Response:** We have indicated the reference we used for this sentence.

Availability of data

**Response:** All data required for this research are available within the manuscript. If additional data are needed, it can be obtained from the corresponding authors upon request.

**Reviewer #2:** Thank you so much for all your valuable and constructive comment.

The objectives of the study are clearly stated and the study design is appropriate to address it. the sample size is appropriate according to WHO standard. The statistical analysis is appropriate to address the objectives.

**Response:** We thank you for your positive feedback to our work.

Reviewer #2: The results are clear and completely presented with tables.

**Response:** Again thank you for your feedback.

Reviewer #2: the conclusion stems from the study findings. There is no limitation of analysis described. The study finding is helpful to advance the understanding of the topic under study and has public health importance.

**Response:** Thank you for your positive comment about our analysis, conclusion and the public health importance of our study.

Reviewer #1: Great work on the revisions. I just have minior comments.  To ensure transparency, please provide the anonymized/coded data you used in the analysis.  Note that policy of PLOS - "The PLOS Data policy requires authors to make all data underlying the findings described in their manuscript fully available without restriction, except in cases where the data are legally or ethically restricted (for example, participant privacy is an appropriate restriction)" The ethical restriction should not be an issue if the each participant is coded.

**Response:** Thank you for your positive feedback to our work.

**Reviewer #3:** Thank you so much for all your valuable and constructive comment. We have accessed your attachment act accordingly.

My main suggestions and recommendations I sent in an attached file. From what I observed in the answers of the authors, they did not access this file. As for the other suggestions made in the general questionnaire, I fully agree

**Response:** Thank you. We have tried to address all your concerns.

**GENERAL CONSIDERATIONS AND SUGGESTIONS**

**Methodology**
It is necessary to clarify the origin of the control group because it is a little strange to have a control group chosen within a hospital. The criterion used to determine the participants in this group has been based only on clinical information as mentioned in the item Socio-demographic characteristics and clinical data? Stool tests has been done? If yes, which method has been used?

**Response:** Thank you for your comment. With regard to sample collection, we took the sample the study participants when they come to the hospital for seeking service. Both stool and blood samples were collected from the eligible participants following systematic random sampling technique. The stool and blood samples for control groups were collected from Dessie blood bank. So, control groups were not from the hospital. All the necessary laboratory and clinical investigations were done for the control groups to include them in the study. Stool examination were done using Kato-Katz technique.

The authors only mention (in the introduction) that schistosomiasis mansoni is endemic in Haik town, not revealing this endemicity index, an important factor in the analysis and determination of the groups. A more detailed description of the area from which these patients originated was lacking. It is important to know if the region has S. haematobium, for example, and if this was also used as an exclusion criterion.

**Response:** Thank you we added some information about the prevalence S. mansoni in Haike town. S. haematobium was not reported from Haike town. As a result we didn’t collect urine sample for the detection of S. haematobium for screening purpose.

In the Item “Stool sample collection and processing”, the last period about blood collection must be removed and added in the following item – “Blood sample collection processing

**Response:** We made the necessary correction.

Patients with splenomegaly were excluded and those with hepatomegaly, too? If these conditions are present in patients with severe forms of schistosomiasis, wouldn't it be interesting to keep them in the study group? An association could be made between the presence of these clinical forms and the hematological and biochemical indices analyzed.

**Response:** Thank you for your comments. You are correct in addition to the hematological and biochemical parameters, imaging techniques especially in chronic infection is very important for monitoring and management of the disease. In this study we could not use the imaging techniques to screen individuals for hepatomegally and splenomegaly. This was indicated in the manuscript as a limitation. We excluded just those with splenectomy based on clinical history not those with splenomegaly and hepatomegaly.

**Results.**
The results are clear and well-presented between tables and graphs, I suggest, however, that you add the standard deviation in the graphs.

**Response:** Thank you. We think that standard deviation is not applicable for the variables in the graph. We just determine the prevalence and severity of anemia among the S. mansoni infected adults.
Discussion
The discussion in relation to hematological and biochemical indices is well founded, however I missed the approach on clinical and socio-demographic data. There was a lack of an approach to public health using indicators from the region and also, the limitations found.

**Response:** Thank you for your comment. We tried to address some of your concerns.
